# Defensive tactics: lessons from *Drosophila*

**DOI:** 10.1242/bio.061609

**Published:** 2024-12-24

**Authors:** Madhumala K. Sadanandappa, Subhana Ahmad, Robinson Mohanraj, Mrunal Ratnaparkhi, Shivaprasad H. Sathyanarayana

**Affiliations:** ^1^ Laboratory for Clinical Genomics and Advanced Technology, Department of Pathology and Laboratory Medicine, Dartmouth-Hitchcock Medical Center, Lebanon, NH 03756, USA; ^2^ lndependent Scholar; ^3^ Biomedical Science, Nitte University for Science Education and Research, Mangalore, Karnataka 575018, India

**Keywords:** Host-parasitoid, Pre-infection behaviors, Innate response, Anti-parasitoid behaviors, Multimodal inputs, Evolutionary arms race

## Abstract

Parasitoid wasps exert strong selective pressure on their hosts, driving the evolution of diverse defense strategies. *Drosophila*, a widely studied model organism, hosts a wide range of parasites, including parasitoid wasps, and has evolved immune and behavioral mechanisms to mitigate the risk of parasitization. These defenses range from avoidance and evasion to post-infection immune responses, such as melanotic encapsulation. In response, parasitoid wasps have developed countermeasures, contributing to an ongoing arms race between host and parasite. This article reviews the anti-parasitoid behaviors of *Drosophila*, focusing on their role in reducing parasitization and enhancing host survival and fitness. It also explores the molecular and neuronal circuit mechanisms that underlie these behaviors, using *Drosophila* as an ecologically relevant model for studying host-parasitoid interactions. Furthermore, the article discusses the potential applications of these findings in biological pest control and highlights key unresolved questions in the field.

## Teaser

**Summary:** This Review explores the *Drosophila* host-parasitoid system as a powerful model for advancing our understanding of hostparasitoid interactions.

## Introduction

Parasitoid wasps, which belong to various hymenopteran superfamilies, represent a vast and diverse group of obligate parasitic insects. With an estimated 350,000 species in the wild, these wasps exert intense selective pressure on their hosts (Gaston, 1991). In response, hosts have evolved various pre- and post-infection defenses, including immunological responses, physiological adaptations, and behaviors such as evasion, avoidance, wriggling, kicking, and camouflage, to reduce the risk of parasitization. In turn, parasitoid wasps have developed counter-adaptations to circumvent these host defenses, resulting in a dynamic evolutionary arms race (Cinel et al., 2020; Ezenwa et al., 2022; Lopes, 2023; Wertheim, 2022). The emerging understanding of the cellular and molecular mechanisms underlying host defenses and parasitoid countermeasures offers insights into ecological and evolutionary dynamics, with important implications for the use of parasitoid wasps in biological pest control.

## *Drosophila* parasitoids

*Drosophila* species host a wide range of parasites, including viruses, bacteria, fungi, nematodes, and parasitoid wasps, which profoundly impact their physiology, behavior, and fitness (Grenier and Leulier, 2020; Montanari and Royet, 2021; Wallace and Obbard, 2024). For example, the protozoan parasite *Crithidia* targets the gut, impairing digestive health and immune responses (Boulanger et al., 2001, 2006). The *Drosophila C Virus* (DCV), a positive-strand RNA virus, also infects the gut, significantly reducing the host's fitness by inducing paralysis or early mortality (Gomariz-Zilber et al., 1995; Huszar and Imler, 2008). The intercellular bacterial parasite *Wolbachia* manipulates host reproduction through mechanisms, such as cytoplasmic incompatibility and germ stem cell proliferation, promoting its transmission (Fast et al., 2011; Min and Benzer, 1997; Serga et al., 2023). The fungal pathogen *Entomophthora muscae* triggers summit disease, where infected flies (often called ‘zombie flies’) climb to elevated surfaces before dying, facilitating fungal spore dispersal (Cohn, 1855; Elya et al., 2018, 2023). Furthermore, the nematode *Howardula aoronymphium* infects the reproductive system, causing sterility in female flies (Haselkorn et al., 2013; Jaenike and Perlman, 2002). These diverse and complex parasitic interactions and the availability of comprehensive connectome maps and advanced genetic toolkits make *Drosophila* a powerful model for studying immunity, behavioral adaptations, and evolutionary biology under laboratory conditions.

**Fig. 1.**
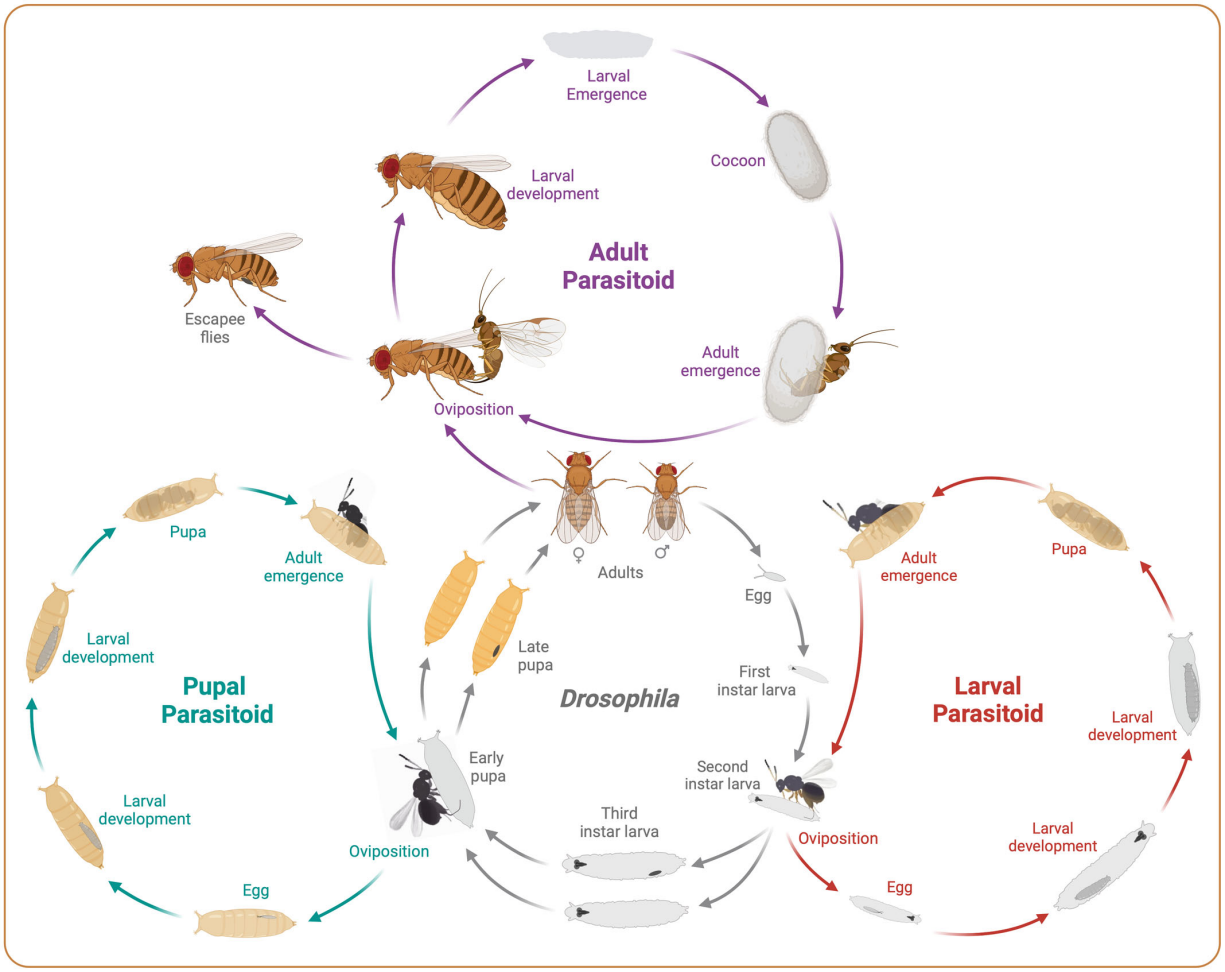
**Life cycles of *Drosophila* and parasitoid wasps.** The schematic illustrates the life cycle of *Drosophila* (the host, shown in grey) alongside its larval (red), pupal (dark cyan), and adult parasitoids (purple). After the wasp lays its eggs inside the host, one of two outcomes occurs: either the host's immune response halts the wasp's development through melanotic encapsulation (visible as black spots in the infected host), leading to the emergence of resistant flies (escapees), or the wasp suppresses the host's immune defenses and successfully completes its life cycle.

*Drosophila* parasitoids belong to four superfamilies of Hymenoptera: Chalcidoidea, Cynipoidea, Ichneumonoidea, and Diaprioidea. These groups have independently evolved to parasitize various *Drosophila* species, employing distinct parasitic strategies (Lue et al., 2021). For over two centuries, it has been established that these parasitoid wasps typically target *Drosophila*’s larval or pupal stages. However, a recent discovery has identified adult-stage parasitoids known as imagobionts, belonging to the Braconidae family (subfamily Euphorinae, genus *Syntretus*) that specifically parasitize adult *Drosophila* (Moore et al., 2024) (Fig. 1).

Larval parasitoids from the Branconidae (*Asobara*) and Figitidae (*Leptopilina*, *Ganaspis*) families are koinobionts, allowing the host to continue its development while the parasitoid wasp develops within it. In contrast, pupal parasitoids from the Diapriidae (*Trichopria*), Pteromalidae (*Pachycrepoideus*), and Encytidae families are idiobionts, which arrest host development immediately after parasitization (Carton et al., 1986; Lue et al., 2021; Prévost, 2009).

*Drosophila* parasitoids oviposit their eggs inside the host, where the eggs develop (Carton et al., 1986; Prévost, 2009). Female parasitoids use specialized ovipositors located at the abdominal tip to inject fertilized or unfertilized eggs, along with immune-suppressive factors such as virus-like particles or venom, into the host's hemolymph (Dupas et al., 1996; Fleury et al., 2009; Gueguen et al., 2011). In response to parasitization, the host mounts a complex immune response characterized by melanotic encapsulation. This defensive cellular mechanism mediated by the lymph gland and the hemocytes, where activated plasmatocytes and lamellocytes aggregate to form a multilayered capsule around the parasitoid egg, eventually killing it (Carton et al., 2008; Nappi and Vass, 1993; Russo et al., 1996) (Fig. 1). Resistant hosts that successfully develop into adult flies, known as ‘escapee flies’, can be identified by the melanized capsules visible through the abdominal cuticle or upon dissection (Keebaugh and Schlenke, 2014; Lemaitre and Hoffmann, 2007; Rizki and Rizki, 1984; Rizki et al., 1990; Small et al., 2012). If the host's immune system fails to encapsulate the deposited egg, the parasitoid larva hatches and progresses through its preimaginal stages, feeding on the host's internal tissues before emerging as an adult from the *Drosophila* pupal case (Carton and Nappi, 1997) (Fig. 1).

Recently, *Syntretus perlmani*, a previously undescribed euphorine species, was found to parasitize adult *Drosophila* species, including *D. melanogaster* and *D. affinis* (Moore et al., 2024). After oviposition, the wasp's larva develops within the active host and eventually emerges by chewing through the abdominal cuticle. The larva then forms a cocoon beneath a loose substrate, completing its development into an adult wasp that emerges from the cocoon (Moore et al., 2024) (Fig. 1).

This remarkable diversity of parasitoid strategies, combined with their wide range of *Drosophila* hosts, makes this system an exceptional model for studying host-parasitoid interactions in laboratory settings (Mauthner et al., 2024; Sadanandappa and Bosco, 2024b; Sadanandappa et al., 2023; Small et al., 2012). Research involving *Drosophila* and its parasitoids, including experiments conducted aboard the International Space Station (ISS) during SpaceX-14's mission (Chou et al., 2024), has provided valuable insights into ecological and evolutionary dynamics, with applications extending into biological control research.

**Fig. 2.**
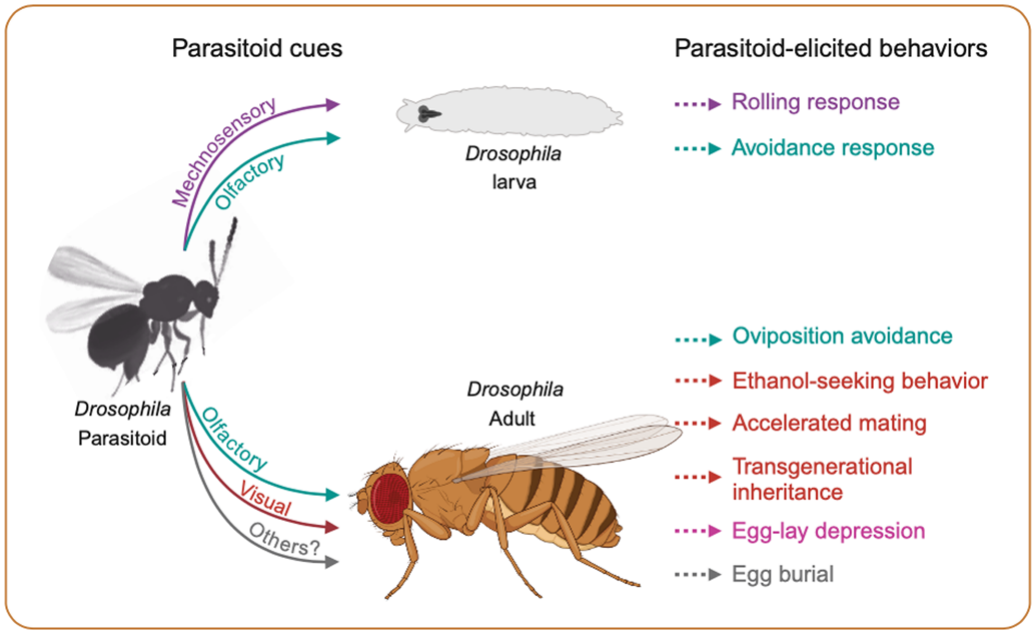
**Anti-parasitoid behaviors of *Drosophila.*** This schematic depicts the pre-infection behaviors displayed by *Drosophila* larvae and adults to defend against parasitoids, along with the sensory mechanisms that underlie these responses. For additional details, refer to the main text and Table 1.

This Review focuses explicitly on the anti-parasitoid behaviors exhibited by *Drosophila* larvae and adults, such as evasion, avoidance, and reproductive modifications (Fig. 2). These behaviors complement immune responses and post-infection strategies. that have been documented in previous studies (Bernardo and Singer, 2017; Carton et al., 2008; Davis and Schlenke, 2022; de Roode and Lefèvre, 2012; Parker et al., 2011). Collectively, this body of research deepens our understanding of the complex interactions between *Drosophila* and their parasitoids, offering broader implications for both fundamental and applied research.

**Table 1.**
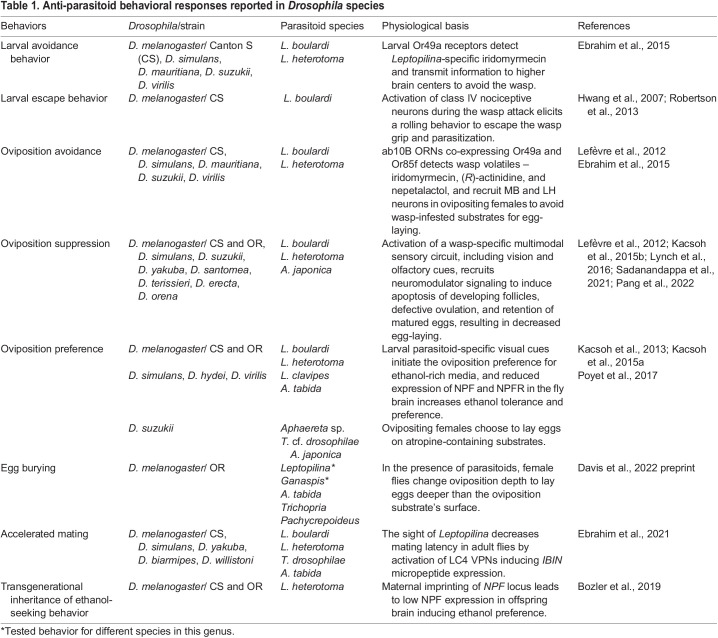
Anti-parasitoid behavioral responses reported in *Drosophila* species

## Larval anti-parasitoid behaviors

Despite their high vulnerability and limited ability to escape parasitoid attacks, *Drosophila* larvae show remarkable resilience when serving as hosts for various parasitoid species in natural environments. Among these parasitoids, *Leptopilina heterotoma* and *Leptopilina boulardi* are the most prevalent, parasitizing a range of *Drosophila* species (Bouletreau et al., 1991; Fleury et al., 2004; Prévost, 2009). In response to parasitization, the larvae initiate cellular immune responses (Carton et al., 2008; Nappi and Vass, 1993; Russo et al., 1996) and may adjust their feeding behavior (Davis and Schlenke, 2022; Milan et al., 2012). For example, parasitized larvae actively seek out alcohol, a secondary metabolite produced by yeasts that grow on decaying food substrates. While *Drosophila* larvae have evolved a tolerance to alcohol, increased ethanol consumption is toxic to the wasp larvae developing within their hemolymph (Milan et al., 2012).

In addition to these post-infection defenses, *Drosophila* larvae display various anti-parasitoid behaviors as a pre-infection defense to mitigate the risk of parasitoid attack, which are further detailed below (Fig. 2 and Table 1).

### Avoidance behavior

*Drosophila* larvae exhibit an ability to avoid parasitoid-infested areas through chemosensory mechanisms. Odor choice behavioral assays demonstrate that *D*. *melanogaster* larvae specifically avoid parasitoid wasps from the genus *Leptopilina*, while displaying no avoidance toward *Asobara* and *Trichopria* (Ebrahim et al., 2015). This innate avoidance of *L. boulardi* and *L. heterotoma*, two common endoparasitoids with different host ranges and virulence strategies (Carton et al., 1986; Fleury et al., 2004; Quicray et al., 2023; Schlenke et al., 2007), is mediated by volatile cues emitted by *Leptopilina* wasp (Ebrahim et al., 2015).

*Drosophila* larval olfactory receptor neurons (ORNs) expressing the olfactory receptor (OR) Or49a detect (−)-iridomyrmecin, a defensive allomone, and sex pheromone component of *Leptopilina* (Stökl et al., 2012; Weiss et al., 2013). This sensory information from ORNs is transmitted to higher brain centers, triggering an avoidance behavior in the larvae. Notably, the olfactory circuit activation in response to iridoid compounds is necessary and sufficient for detecting and avoiding *Leptopilina* wasps, and this mechanism is conserved across several *Drosophila* species (Ebrahim et al., 2015).

### Escape behavior

When attacked by a parasitoid wasp, *Drosophila* larvae display a nocifensive escape behavior characterized by rapid rolling along their anterior-posterior axis (Borjon et al., 2024; Hwang et al., 2007; Mauthner et al., 2024; Robertson et al., 2013). Typically, *D. melanogaster* larvae display peristaltic movements during foraging. However, the penetration of a wasp ovipositor into the larval cuticle triggers an immediate rolling response. This defensive action helps the larvae escape the wasp's grip, sometimes even entangling or flipping the wasp, leading to a premature termination of the attack. After the rolling behavior, the larvae crawl away as the wasp retracts its ovipositor (Hwang et al., 2007).

This escape behavior is particularly effective against inexperienced wasps – those that have not previously oviposited – compared to their experienced counterparts (van Lenteren et al., 1998). The success of this defensive rolling behavior, a well-characterized and conserved locomotory response, depends on the specific location of the wasp's attack and the depth of the ovipositor's penetration into the larval cuticle (Cooney et al., 2023; Robertson et al., 2013; Zhu et al., 2023).

*Drosophila* escape response is mediated by multi-dendritic class IV neurons, which function as primary nociceptors distributed throughout the larval body wall. Each larval hemi-segment contains three identifiable class IV neurons located in dorsal (ddaC), ventral (vdaB), and lateral (v'ada) regions (Grueber et al., 2002). The penetration of the wasp ovipositor into a single nociceptive dendritic field, particularly at high frequencies of cuticle penetration, stimulates the class IV neurons expressing the *pickpocket* gene. Activation of these neurons is necessary and sufficient to induce rolling behavior in response to *L. boulardi* attacks (Hwang et al., 2007; Robertson et al., 2013). Thus, the mechanosensory stimulus provided by the parasitoid attack plays a crucial in eliciting nociceptive behavior. However, further investigation is needed to determine whether this response is generalized to defend against other wasp species.

## Adult anti-parasitoid behaviors

Until recently, parasitoid wasps targeting *Drosophila* were known to infect fruit fly's progeny−larval or pupal stages. However, the recent discovery of a parasitoid wasp capable of infecting adult-stage *Drosophila* expands our understanding of host-parasitoid interactions (Moore et al., 2024). Remarkably, adult flies have developed sensory modalities, such as vision, olfaction, gustation, and audition, which allow them to detect and distinguish between potential parasitoid threats. Upon sensing parasitoid wasps, multimodal sensory inputs activate a cascade of behavioral and physiological modifications in *Drosophila*, reducing the risk of wasp infection in their offspring, which remain highly vulnerable to parasitoid attacks due to their limited defense mechanisms (Davis and Schlenke, 2022; Lopes, 2023).

*Drosophila* adults display sophisticated defenses against parasitoid threats, including avoiding wasp-infested food substrates, selecting safer oviposition sites, protecting their progeny during wasp attacks, and even communicating threat signals to other flies (Table 1) (Davis and Schlenke, 2022; Montanari and Royet, 2021). These protective behaviors serve beyond immediate survival purposes by influencing offspring defense behaviors over multiple generations, highlighting the significance of parental response to parasitoid pressure (Bozler et al., 2019; Bozler et al., 2020; Khan et al., 2023 preprint; Singh et al., 2015; Tetreau et al., 2019).

### Oviposition avoidance behavior

Unlike larvae, *Drosophila* adults do not show direct repulsion to *Leptopilina* odors in behavioral choice assays, such as T-maze and trap assay. However, ovipositing females avoid sites infested with *Leptopilina* wasps when laying eggs. This innate oviposition avoidance behavior is mediated by Or49a and Or85f receptors, co-expressed in antennal ab10B ORNs (Couto et al., 2005). These ORNs detect parasitoid-derived volatile compounds, such as iridomyrmecin, (*R*)-actinidine, and different enantiomers of nepetalactol, alerting the flies to parasitoids presence. Upon activation, these antennal ORNs relay sensory information to higher brain centers, including the mushroom body (MB) and lateral horn (LH). The subsequent neuronal processing leads to the avoidance of *Leptopilina*-infested oviposition substrates, thereby minimizing the risk of parasitization and enhancing the survival rate of their progeny. This anti-parasitoid behavior is a conserved feature across several *Drosophila* species (Ebrahim et al., 2015; Lefèvre et al., 2012) (Table 1).

### Oviposition suppression

In the presence of *Leptopilina* wasps, ovipositing *Drosophila* females significantly reduce egg-laying as a defensive measure to safeguard their offspring from parasitism. This *Leptopilina*-selective reproductive modification, documented across several *Drosophila* species, illustrates the complex and diverse behavioral adaptations female flies employ in response to different *Leptopilina* species (Kacsoh et al., 2015b; Lefèvre et al., 2012; Lynch et al., 2016; Pang et al., 2022; Sadanandappa et al., 2021).

Visual detection of *L. heterotoma* induces a prolonged suppression of egg-lay in *Drosophila* females. This reproductive modification results from increased apoptosis in developing egg chambers, reducing ovary size. Unlike the innate suppression that occurs in the presence of wasps, a learned suppression persists even after the wasps are removed, requiring inputs from MB neurons and the expression of learning and memory-related genes, such as *rutabaga*, *dunce*, *Adf1*, *dFmr1*, *amnesiac*, and *Orb2*. Parasitoid-exposed females can communicate this threat information to naïve flies, both within the same species (intra-species) and across different species (inter-species), further reducing egg-laying (Kacsoh et al., 2015b, 2018).

In response to *L. boulardi*, a transient reduction in egg-laying is triggered by multimodal sensory cues involving visual and olfactory (Or49a and Or85f) stimuli (Lynch et al., 2016; Sadanandappa et al., 2021). Activation of these integrated circuits recruits neuroendocrine signaling through neuropeptide F (NPF) and its receptor, NPFR, mediating egg-laying suppression. This behavioral response involves apoptosis in the developing follicles and retention of the matured egg chambers, which are laid later in wasp-free oviposition sites (Sadanandappa et al., 2021, 2023).

Interestingly, the effectiveness of the parasitic attack, which declines with wasp age, influences the degree of egg-lay reduction. Younger *Leptopilina* wasps exhibit a more aggressive host-seeking behavior, which *Drosophila* females detect through LC4 cells, a class of visual projection neurons (VPN) in the lobula region of the visual center. This sensory information is relayed to higher brain centers, reducing octopaminergic signaling to the female germline, disrupting ovulation, and causing retention of matured follicles, eventually suppressing egg-laying (Pang et al., 2022). In the presence of highly efficient parasitoids, females thus modify their egg-laying timing rather than location, minimizing the threat to their offspring.

### Oviposition preference

When faced with parasitoid threats, *Drosophila* females halt energy-intensive egg production and reallocate their resources to assess oviposition sites free of wasps. This defensive behavior aims to either prevent parasitization (anti-parasitization strategy) or provide optimal conditions for offspring with enhanced immunity (medicative strategy) (Aranha and Vasconcelos, 2018; Bozler et al., 2020; Montanari and Royet, 2021).

For instance, in the presence of female larval parasitoid wasps, *Drosophila* females preferentially lay their eggs on media containing toxic levels of alcohol. This anti-parasitization strategy repels parasitoids, particularly generalist species, thereby reducing the parasitization rate. Compared to parasitoid larvae, higher ethanol tolerance of fly larvae leads to a significant increase in the survival rate of *Drosophila* offspring (Lynch et al., 2017; Milan et al., 2012). This innate ability of female flies to recognize parasitoid threats and their preference for ethanol-enriched media is mediated by visual cues and reduced expression of NPF and NPFR in the brain (Kacsoh et al., 2013). However, prolonged parasitoid exposure induces persistent changes in ethanol-seeking behavior, which requires synaptic transmission by MB neurons and the functioning of learning and memory-related genes, such as *rutabaga*, *amnesic*, *dunce*, *Orb2*, and *dFmr1* (Kacsoh et al., 2015a).

Similarly, the spotted-wing *Drosophila*, *D. suzukii*, an invasive species in the United States and Europe originating from Asia, adopts a medicative strategy when threatened by parasitoids (Asplen et al., 2015; Calabria et al., 2012). Upon detecting larval (*T. drosophilae*) and pupal parasitoids (*A. japonica*), *D. suzukii* females prefer laying eggs on food containing atropine, an entomotoxic alkaloids found in fruits of *Atropa belladonna* (commonly known as deadly nightshade). While adult parasitoid wasps are not repelled by atropine, infected *D. suzukii* larvae on atropine-enriched substrates exhibit enhanced resistance, resulting in higher survival rates. Moreover, parasitoids emerging from host larvae developed on atropine-containing media display reduced fitness, including delayed developmental time, smaller body size, and lower reproductive efficiency (Poyet et al., 2017). Further understanding of the cellular and molecular mechanisms that drive *Drosophila*’s detection of different parasitoid species and their oviposition preferences may contribute to better biocontrol strategies for *D. suzukii*.

### Oviposition depth

In addition to selecting suitable timing and location for egg-laying, ovipositing *Drosophila* adult flies adjust their oviposition depth in response to parasitoid threats. Female flies lay eggs deeper within the food substrate, particularly when exposed to larval parasitoids such as *Leptopilina*, *Ganaspis*, and *Asobara*, or pupal parasitoids like *Pachycrepoideus* and *Trichopria* (Davis et al., 2022 preprint). This anti-parasitoid behavior, observed across the *Drosophila* genus, protects the eggs by embedding them below the surface, reducing risks from parasitoids and ant predation. However, whether this adaptive behavior specifically deters parasitoids or is a broader response to hymenopteran cues remains unclear.

### Accelerated mating

*Drosophila* adults exhibit an adaptive acceleration of mating behavior in the presence of parasitoid wasps (Ebrahim et al., 2021). This heightened sense of urgency ensures that flies engage in quicker courtship and mating, allowing them to conserve time and resources for critical activities, such as searching for parasitoid-free oviposition sites for egg-laying. Species like *L. boulardi*, *L. heterotoma*, *T. drosophilae*, and *A. tabida*, which parasitize *Drosophila*, trigger this mating acceleration in various *Drosophila* species, including *D. melanogaster*, *D. simulans*, *D. yakuba*, *D. biarmipes*, and *D. willistoni*. Although the precise neuronal circuits involved remain unclear, the sight of parasitoids has been shown to activate LC4 VPNs, which leads to the expression of the IBIN (*Induced by Infection*) micropeptide, reducing mating latency (Ebrahim et al., 2021). This rapid courtship behavior is crucial for ensuring reproductive success in environments with a high risk of parasitization.

### Transgenerational inheritance

In *Drosophila*, parental exposure to parasitoids primes offspring to mount rapid and more efficient immune responses, resulting in higher survival rates following parasitic infection. Strikingly, this heightened immune response is not dependent on parents themselves being infected, suggesting that parasitoid-exposed parental flies transmit immune-priming traits to their offspring through intergenerational and transgenerational inheritance. This demonstrates that *Drosophila* adults make strategic reproductive decisions, laying eggs that are genetically predisposed to resist parasitoid attacks (Bozler et al., 2020; Khan et al., 2023 preprint; Singh et al., 2015; Tetreau et al., 2019).

In addition to immune priming, prolonged exposure to parasitoids influences ethanol-seeking behavior in offspring for up to five generations (Bozler et al., 2019; Kacsoh et al., 2013). Upon encountering visual cues from *L. heterotoma*, reduced NPF expression in the maternal brain triggers a shift in oviposition behavior and induces caspases-mediated apoptosis in developing follicles, suppressing egg-laying. These activated effector caspases facilitate epigenetic reprogramming of the maternal germline, resulting in both male and female progeny inheriting ethanol preferences that are passed down to subsequent generations. This preference is driven by reduced NPF expression in the fan-shaped body of female offspring imprinted at the NPF locus results (Bozler et al., 2019). These findings support the transgenerational inheritance of behaviors in invertebrate models, an emerging area of research that challenges traditional views on inheritance and behavior.

## Future perspectives

The dynamic interplay between host defense mechanisms and parasitoid counteroffensive strategies, particularly in response to newly evolved host defenses, offers a compelling study area within host-parasitoid systems. The *Drosophila* model, instrumental in biomedical research, also provides an ecologically relevant framework for investigating these interactions (Bellen et al., 2010; Davis and Schlenke, 2022; Mohr, 2018). Its sophisticated genetic tools, comprehensive neural connectome, and the diversity of its parasitoid species – each with distinct host ranges and virulence strategies – make it an ideal system for studying the complexities of host defenses.

This Review highlights pre-infection behavioral defenses, where *Drosophila* larvae and adults exhibit threat detection, avoidance, and escape behaviors. These proactive strategies emphasize the importance of early defensive measures in reducing parasitoid success. Post-infection defenses, such as dietary self-medication, also contribute to host survival, although the full spectrum of behavioral modifications remains to be explored (Vale et al., 2018). Conversely, parasitoid employ countertactics to manipulate host behavior, altering physiology and actions to enhance their own survival (Sadanandappa and Bosco, 2024a; Zhang et al., 2021). A systematic exploration of these interactions across various *Drosophila* species and their parasitoids promises to unravel intricate dynamics and defense strategies, contributing to our understanding of behavioral immunity.

At the mechanistic level, we are beginning to identify the roles of sensory systems – vision, olfaction, and mechanosensation – along with higher brain centers like the MB, LH, and fan-shaped body, and the neuromodulator signaling involving NPF and octopamine in orchestrating host defenses (Davis and Schlenke, 2022; Montanari and Royet, 2021). However, much remains to be explored, such as how different sensory modalities, including gustation and audition, integrate to elicit a specific behavioral response. Additionally, understanding how hosts differentiate between different parasitoid species and fine-tune their defenses is a prime area of research.

From an evolutionary perspective, the trade-offs between behavioral and physiological adaptations in host defenses still need to be fully understood. At the same time, parasitoids evolve their own strategies to circumvent host defenses and enhance their fitness (Pang et al., 2024; Sadanandappa and Bosco, 2024a; Sheng et al., 2023; Zhang et al., 2023). Beyond laboratory studies, future field-based research will be essential to uncover how these coevolutionary dynamics play out in natural environments, providing insights into how hosts and parasitoids adapt to each other over time.

The ecological and agricultural implications of this research are particularly relevant to pest management. Understanding the host-parasitoid interactions in *Drosophila* offers the potential for improving biological control methods, especially for managing *D. suzukii*, an invasive agricultural pest (Lee et al., 2019; Walsh et al., 2011). While parasitoid wasps show promise as biocontrol agents, their efficacy is often limited in non-native regions where *D. suzukii* has not co-evolved with local parasitoids. Introducing co-evolved parasitoids from the pest's natural range may offer a more effective, sustainable alternative to chemical pesticides, reducing environmental impact, promoting biodiversity, and fostering healthier ecosystems (Girod et al., 2018a,b; Kruitwagen et al., 2018).

In conclusion, the *Drosophila* host-parasitoid system is a powerful model for advancing our understanding of host-parasitoid interactions. Ongoing research in this area will continue to illuminate the complexities of behavioral and physiological immunity while offering practical applications in sustainable pest control and agricultural management.
